# Effects of Upper Eyelid Blepharoplasty on Contrast Sensitivity in Dermatochalasis Patients

**DOI:** 10.4274/tjo.galenos.2019.95871

**Published:** 2020-06-27

**Authors:** Hilal Nalcı, Melek Banu Hoşal, Ömür Uçakhan Gündüz

**Affiliations:** 1 Ankara University Faculty of Medicine, Department of Ophthalmology, Ankara, Turkey

**Keywords:** Blepharoplasty, contrast sensitivity, dermatochalasis, high order aberration, keratometry, pseudoptosis

## Abstract

**Objectives:**

To evaluate the impact of upper eyelid blepharoplasty on contrast sensitivity in dermatochalasis patients.

**Materials and Methods:**

Best corrected visual acuity, ophthalmologic examination, eyelid examination, lash ptosis, contrast sensitivity using sine-wave contrast sensitivity chart, keratometric parameters, and corneal aberrations of 34 eyes of 34 patients who underwent upper eyelid blepharoplasty due to dermatochalasis in our clinic between the years 2014 and 2018 were evaluated preoperatively and at postoperative 3 months.

**Results:**

Twenty-three (68%) of the patients were females and 11 (32%) were males. Mean age was 63.1±7 (52-81) years. Mean best corrected visual acuity was 0.036±0.06 (0-0.15) logMAR preoperatively and postoperatively (p>0.05). Contrast sensitivity values of the patients at the frequencies of 1.5, 3, 6, 12, and 18 cycles per degree were 44.38±19.5, 59.03±27.2, 41.44±34.1, 15.15±19.3, and 5.15±4.26 preoperatively and 44.80±20.9, 76.85±33.4, 63.21±46.4, 28.21±31.1, and 10.5±9.5 postoperatively, respectively. The difference between contrast sensitivity values was statistically significant at the frequencies of 3, 6, 12, and 18 cpd (p=0.005, =0.001, <0.001, and <0.001, respectively). Although lash ptosis of the patients improved significantly after the surgery, there was no correlation between lash ptosis improvement and change in contrast sensitivity (p>0.05). Keratometric values and corneal high order aberrations did not change significantly after the surgery (p>0.05).

**Conclusion:**

Contrast sensitivity significantly increases after upper eyelid blepharoplasty, especially at higher spatial frequencies which are known to deteriorate due to age-related changes in the lens and retina in older adults. Our results show that blepharoplasty may have additional functional indications for elderly dermatochalasis patients in terms of improving the functions such as performing daily tasks and reading.

## Introduction

Dermatochalasis is an age-related condition characterized by the development of a fold of excess skin over the upper eyelid with loss of skin elasticity. It is sometimes accompanied by herniation of orbital adipose tissue through the orbital septum, which also weakens with age.^1^ The mechanical pressure created by the accumulation of excess adipose and skin over the upper eyelid leads to a feeling of heaviness and narrowing of the peripheral visual field, constituting an indication for surgical treatment.^2,3^

The increase in the visual field after upper eyelid blepharoplasty is known to provide a functional benefit to patients. In addition, Rogers et al.^4^ also observed that these patients reported a subjective increase in vision postoperatively. Although the authors did not observe an objective change in refraction tests, they detected improvement in the patients’ contrast sensitivity test results. Similar results were also obtained by An et al.^5^ and Fowler et al.^6^ The exact reason for the postoperative increase in contrast sensitivity is unclear. Proposed explanations include changes in higher-order aberrations (HOA), elimination of the hooding effect created by the eyelids and eyelashes, or changes in corneal topography and keratometry.^5,6,7,8^

The aim of this study was to observe changes in contrast sensitivity in patients who underwent upper eyelid blepharoplasty due to dermatochalasis and to investigate the potential causes of these changes.

## Materials and Methods

Patients diagnosed with dermatochalasis between 2014 and 2018 in the oculoplasty unit of the Ankara University Faculty of Medicine, Department of Ophthalmology were examined. Surgery was indicated for patients with a narrowed upper visual field in automated perimetry and for those reporting complaints of visual field narrowing even if they were unable to comply with the test. Patients with an accompanying eyelid condition or history of previous eyelid surgery and those with brow ptosis, dry eye, or any lens, optic nerve, or retinal pathologies were excluded from the study. The study was carried out in accordance with the Declaration of Helsinki and approval was obtained from the Ethics Committee of Ankara University Faculty of Medicine.

All operations were performed by a single surgeon (B. H.). During the procedure, the excess skin tissue overhanging the upper eyelid was excised; adipose tissue excision was not performed in any of the patients. All patients’ best corrected visual acuity (measured with Snellen chart and presented in logMAR equivalents), ocular surface examination findings, tear film break-up times, Schirmer’s test results, and upper eyelid examination findings were recorded preoperatively and at postoperative 3 months. Upper eyelid lash ptosis was graded by a single physician through clinical examination and photograph analysis. Grading was classified as none (0), mild (1), moderate (2), or severe (3) based on evaluation of lash position relative to the lid margin in primary and lateral positions (Table 1).^5^ Keratometric parameters and corneal HOAs were recorded using a Scheimpflug system (Pentacam, Oculus, GmBH, Germany). Contrast sensitivity was measured with a sine-wave contrast test (Stereo Optical Co., Inc., USA) preoperatively and at postoperative 3 months. Using this chart, contrast sensitivity was evaluated at 5 spatial frequencies: 1.5, 3, 6, 12, and 18 cycles per degree (cpd). After correcting refractive errors, the chart was placed at a standard distance (3 meters) in an environment with standard photopic illumination and the patients were asked to indicate the orientation of the stripes in the circles on the chart as tilted left, tilted right, or vertical. The last degree at which the patients responded correct was marked on the contrast sensitivity curve. Descriptive data were expressed as mean ± standard deviation, median, and minimum-maximum values. After testing the normality of the data distribution, differences between pre- and postoperative results were evaluated using Wilcoxon test. The relationship between postoperative changes in independent variables was evaluated with Spearman’s correlation coefficient. A p value of <0.05 was regarded as statistically significant.

## Results

**Figure 1:**
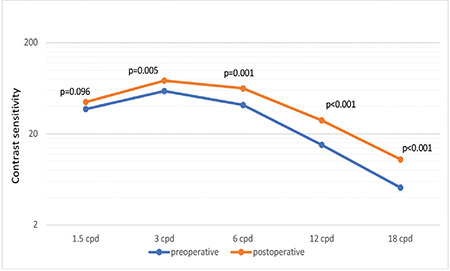
Pre- and postoperative contrast sensitivity values cpd: cycles per degree

Thirty-four patients were included in the study, 23 (68%) women and 11 (32%) men. The average age was 63.1±7 (51-81) years. The mean best corrected visual acuity both pre- and postoperatively was 0.036±0.06 (0-0.15) logMAR. Mean contrast sensitivity percentage values at the frequencies of 1.5, 3, 6, 12, and 18 cpd were 44.38±19.5, 59.03±27.2, 41.44±34.1, 15.15±19.3, and 5.15±4.26 preoperatively and 44.80±20.9, 76.85±33.4, 63.21±46.4, 28.21±31.1, and 10.5±9.5 at postoperative 3 months, respectively. The difference between pre- and postoperative contrast sensitivity measurements was statistically significant at frequencies of 3, 6, 12, and 18 cpd (Wilcoxon p<0.05) (Figure 1). The median preoperative lash ptosis grade was moderate (Grade 2). Postoperatively, lash ptosis improved by at least 1 grade in 20 patients, and the postoperative median lash ptosis grade was mild (Grade 1). The difference between pre- and postoperative eyelash ptosis grade was statistically significant (Wilcoxon p<0.001). The relationship between postoperative change in lash ptosis and change in contrast sensitivity was evaluated with Spearman’s correlation coefficient and no significant relationship was found at any frequency (1.5 cycles per degree [cpd]: rho=0.216, p=0.24; 3 cpd: rho=-0.98, p=0.6; 6 cpd: rho=-0.107, p=0.56; 12 cpd: rho=-0.042, p=0.83, 18 cpd: rho=0.098, p=0.6).

No significant differences were observed between preoperative and postoperative keratometric values, degrees of astigmatism, and corneal HOAs (p>0.05) (Table 2).

## Discussion

Spatial contrast refers to the light/dark transition in the edge or corner of an object or pattern. Contrast sensitivity is a measure of the minimum contrast needed to distinguish a pattern. Visual acuity, on the other hand, measures how large an object must be in order to be seen, and the shapes used in these tests are presented at very high contrast levels. Contrast sensitivity defines the limits of visual perception at different spatial frequencies, and is the factor that determines the visual function of patients having equal visual acuity under conditions with reduced contrast between an object and its background, such as in low or bright light or fog.^9,10^ Similar to previous literature findings, our results demonstrated a statistically significant increase in patients’ contrast sensitivity after upper eyelid blepharoplasty. These results are also consistent with studies in which patients with dermatochalasis reported subjective improvement in contrast sensitivity-related symptoms, such as increased image brightness and comfort in reading and driving, after surgery.^4,5^

In the present study, contrast sensitivity was measured using a sine-wave grating chart, which enabled the evaluation of patients’ contrast sensitivity levels at different spatial frequencies. Loss of sensitivity at high frequencies is known to occur in older adults due to age-related changes such as loss of lens transparency and degeneration of rod photoreceptors in the retina.^11,12,13^ While the mean contrast sensitivity of our patients was found to be below normal limits, especially at frequencies of 6 and 18 cpd, it returned to within normal range postoperatively. Contrast sensitivity has been shown to be associated with visual functions such as vision, reading, and driving in low-light conditions and has been identified as a significant indicator of functional vision.^10^ Therefore, dermatochalasis surgery may have the capacity to partially mitigate the impairment in quality of daily life associated with age-related deterioration of functional vision.

Various mechanisms have been proposed to explain the postoperative increase in contrast sensitivity and visual function. The first is changes in corneal keratometry. There are publications demonstrating changes in corneal astigmatism after levator resection for the treatment of ptosis. This indicates that modifying upper lid position can change corneal refractive power.^14,15^ However, studies on the effects of upper eyelid blepharoplasty on keratometric values present varying results. Brown et al.^14^ reported a mean change of 0.5 D in the astigmatism of these patients. According to Zinkernagel et al.^15^, significant keratometric changes occured after upper eyelid blepharoplasty in patients with severe dermatochalasis who required adipose tissue excision. Şimşek et al.^16^ and Altın Ekin et al.^17^ observed statistically significant changes in astigmatism after surgery, but reported that this difference did not influence visual acuity levels. Doğan et al.^18^ reported that upper eyelid blepharoplasty did not affect keratometric values. Our patients also showed no significant postoperative change in corneal keratometric parameters, and the mean change in astigmatism was 0.06 D. This may be because the operations performed in our clinic did not include adipose tissue excision and there was not a significant change in the pressure exerted by the upper eyelid on the cornea.

Another mechanism proposed as the reason for the increase in contrast sensitivity is the postoperative elimination of the diffraction effect caused by ptotic eyelashes. Kim et al.^7^ suggested that the increase in contrast sensitivity after surgery may have been due to the reduction in lash ptosis that they observed. However, they did not statistically evaluate the relationship between lash ptosis and contrast sensitivity changes in their study. In the present study, we also observed lash ptosis improvement of at least one grade in over half of the patients after surgery, but no relationship was detected between the change in lash ptosis and change in contrast sensitivity. Based on these results, reduced lash ptosis alone cannot explain the increase in contrast sensitivity. In addition to the lash effect, allowing more light to enter the eye by eliminating the hooding effect caused by the overhanging skin fold is another proposed mechanism.^4,5^

Change in HOAs is yet another proposed mechanism for the improvement in contrast sensitivity.^7,19^ Corneal HOAs are refractive disorders that stem from irregularities in the corneal layer and impair the quality of the retinal image.^20^ Dry eye^21^, advanced age^22^, degenerative diseases such as keratoconus^23^, and corneal surgeries that alter corneal curvature^24,25^ have an effect on corneal aberrations.^20,21,22,23,24,25^ There is little information in the literature about the impact of upper lid and lash position on corneal HOAs. Han et al.^26^ showed that excessive skin tissue over the cornea may lead to changes in ocular surface curvature and corneal aberrations, and that lash ptosis may cause an increase in ocular aberrations. Kim et al.^7^ observed a decrease in ocular aberrations following dermatochalasis surgery and associated this finding with the improvement in lash ptosis. However, there are few studies on the effect of surgery on ocular aberrations and their relationship with contrast sensitivity. We detected no significant postoperative changes in corneal HOA measurements in this study. In another study published in 2019 by Altin Ekin et al.^17^, it was reported that a statistically significant change was detected in corneal HOA values based on measurements made 1 month after upper eyelid blepharoplasty. The discrepancy between their results and ours may arise from a difference in the patients’ dermatochalasis grades and the effects of the excess skin over the upper lid on the corneal curvature, or from the difference in the timing of postoperative HOA measurement.

### Study Limitations

In addition, the small number of cases, one of the limitations of our study, may have had an effect on statistical significance. Studies including large patient numbers and evaluating corneal and total ocular aberrations and their relationship with both lash ptosis and contrast sensitivity will better clarify these mechanisms.

## Conclusion

The results of our study show that there may be an additional, functional indication for upper eyelid blepharoplasty in older patients with dermatochalasis in order to facilitate daily life and activities such as reading. The increase in contrast sensitivity in our patients may be a result of removing the excess skin and lashes that cause hooding and diffraction in the visual axis, but further studies with more patients are needed to determine the relationship between them.

## Figures and Tables

**Table 1:**
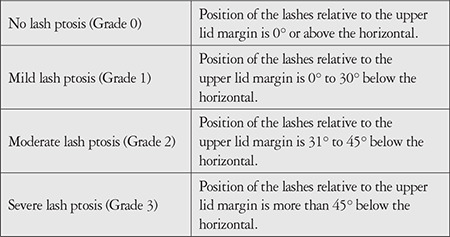
Upper eyelid lash ptosis grading criteria

**Table 2:**
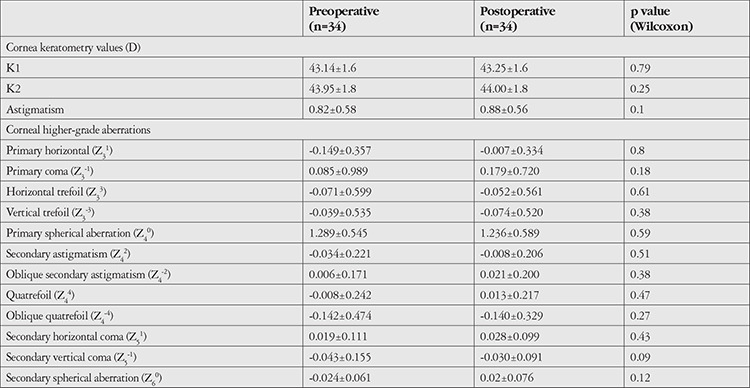
Pre- and postoperative corneal keratometry and corneal aberration values
